# Family matters: health policies to tackle cardiomyopathies across Europe

**DOI:** 10.1093/eurheartj/ehae419

**Published:** 2024-11-16

**Authors:** Iacopo Olivotto, Cardiomyopathies Matter Initiative, Teofila (Tootie) Bueser, Ester Costafreda, Tomás Fajardo, Pablo Garcia-Pavia, Stefan Janssens, Hugo Katus, Stellan Mörner, Iacopo Olivotto, Matteo Pinciroli, Sean Taylor, Maria T Tome Esteban, Patricia Tiramani, Jean-Noël Trochu, Rogier Veltrop, Lee Baker

**Affiliations:** Department of Experimental and Clinical Medicine, University of Florence and Meyer University Children Hospital, Viale Pieraccini 24, 50134 Florence, Italy

## Introduction

Cardiovascular diseases are currently the focus of mounting calls for increased health policy attention in many European countries and European level, being the leading cause of death across Europe and conferring an enormous economic burden—recently estimated at €282 billion annually across the European Union (EU).^1^ That said, not all countries have a national cardiovascular action plan and gaps exist in those that do. While policy efforts rightly place a major focus on the prevention of acquired age-related conditions, cardiomyopathies—i.e. primary myocardial diseases—are often overlooked.

Cardiomyopathies, which are often inherited,^2^ are far from rare with prevalence rates for the most common forms as high as 1/250.^3^ Cardiomyopathies can increase the risk of sudden cardiac death, cause incapacitating symptoms, impair the quality of life of patients and families, and are a leading reason for heart transplantation in the very young. Healthcare costs are often high, especially when patients need to be hospitalized.^4^

New clinical guidelines published by the European Society of Cardiology (ESC) offer an unprecedented opportunity to improve the standards of cardiomyopathy care across Europe.^2^ However, patients’ and families’ access to such care depends heavily on policy and health system factors that can vary between and within countries. These policy factors include support for expert centre networks, screening programmes, and reimbursement of guideline-recommended tests (*Table 1*).

**Table 1:** Landscape of national policies relating to patient care and family screening for inherited cardiomyopathies

Variable	Belgium	France	Germany	Hungary	Italy	Ireland
Relevant, specific national governmental CVD strategy and/or action plan	No	No	No	No	No	No Previous CVD strategy (2010–2019) now lapsed, not replaced
National governmental CVD strategy or action plan with specific actions on inherited cardiac disease such as CM	No Relevant regional initiatives exist, e.g. Vlaams Netwerk Zeldzame Ziekten in Flanders (see here and here)	No	No	No	No	No
Expert centres exist for inherited cardiac diseases/CM (services include genetic testing)	Yes Regional ‘rare disease functions’ exist and expert centres designation in process (see here)	Yes	Yes	Yes (but limited)	Yes	Yes
Inherited cardiac disease/CM expert centres are organized/accredited/linked within a formal nationally/centrally co-ordinated network	No (in process)	Yes Reference centres for hereditary or rare cardiac diseases networked within Cardiogen national referral network for hereditary or rare diseases established by the Ministry of Health, with common protocols for HCM, ARCV, and laminopathies	No	No	Yes Ministry of Health grants the title ‘Istituto di Ricovero e Cura a Carattere Scientifico’ (IRCCS; Scientific Institutes for Research, Hospitalization and Healthcare) to hospitals considered to be a benchmark for patient care quality and innovation (see here). The IRCCS Cardiology Network fosters cooperation between IRCCSs in CVD field information sharing and collaboration	No
National governmental initiatives to promote implementation of/adherence to guidelines for screening and care for CM/inherited cardiac disease among cardiologists	Yes Via the Collège de Cardiologie (organization created by the federal government to promote good-quality CVD care, in collaboration with the Belgian Society of Cardiology) (see here)	Yes Via Cardiogen national HCM, ARCV, and laminopathies protocols	No	No A guideline exists for genetic testing, though not specifically for inherited CVD	No Selected regions have local plans and networks	No
National governmental policy for guideline-led/evidence-based reimbursement of inherited cardiac disease/CM genetic tests (for patients and family members)	Yes Restricted number of accredited human genetics centres where testing is reimbursed by the federal single-payer system. In addition, genetic testing for specialty specific inherited conditions appears to be referred to in the government’s nomenclature of medical treatments (partially) covered by health insurance schemes (see here)	Partially Prescribed genetic tests are reimbursed at 60% by the French social security system Additional (variable) reimbursement from private healthcare companies possible (see here)	Yes Costs of human genetic counselling and tests are covered in full by statutory health insurance funds (e.g. see here, here, here, here, and here)	No Genetic testing is reimbursed on an individual case-by-case basis	Yes Genetic testing is fully covered by the NHS	No
National governmental initiatives to promote inherited cardiac disease/CM family screening (among cardiologists, primary care physicians, nurses, public, etc.)	No Objective of the recent Flemish network of rare diseases	Yes Cardiogen protocols for HCM, ARCV, and laminopathies cover family screening	No	No	No	No
National governmental initiative to promote health literacy and/or shared decision-making in cardiology clinical practice	No	No	No	No	No	No
National governmental initiatives to support networked data sharing infrastructures that include inherited cardiac diseases/CM	No	Yes BaMaCoeur, the Cardiogen database, compiles all data on patients with hereditary or rare heart diseases within the network in a single file	Yes The Federal Research Ministry is financing CAEHR (Cardiovascular Diseases—Enhancing Healthcare through Cross-Sectoral Routine data integration) and ACRIBiS, a multisite project combining standardized and structured clinical records and biosignal analyses to improve personalized CVD risk assessment. Government (BMBF)-funded research networks on common diseases (DZG) and the federated learning activity within the Cardiovascular Research Center (DZHK) also provide data sharing infrastructures	No	No	No
Direct national governmental funding dedicated specifically to CM/inherited CVD projects (within last 5 years)	No	Yes By Cardiogen (see here and here)	Yes Via the German Center for Cardiovascular Research (DZHK) and the German Research Foundation (DFG)	No	Yes The Directorate for Research and Innovation in Health in the Ministry of Health finances specific research projects implemented in IRCCS networks, with resources deriving from the allocation of the National Health Fund (see here). The IRCCS Cardiology Network inherited cardiomyopathies working group aims to implement the adoption of shared clinical and genetic protocols for the precision diagnosis of hereditary cardiomyopathies, the generation of homogeneous groups of cardiomyopathies caused by defects in the same genes, the development of clinical and laboratory research, promoting R&D on new treatments (see here)	No

Variable	Netherlands	Poland	Spain	Sweden	Switzerland	UK
Relevant, specific national governmental CVD strategy and/or action plan	No	Yes National Program on Cardiovascular Diseases 2022–2032 (Narodowy Program Chorób Układu Krążenia; NPChUK)	Yes Estrategia de Salud Cardiovascular del Sistema Nacional de Salud (ESCAV; April 2022)	No	No	Yes CVD plan within NHS England Long-Term Plan (here and here), specific Scottish Heart Disease Action Plan (2021), and Welsh Heart Conditions Delivery Plan (2017)
National governmental CVD strategy or action plan with specific actions on inherited cardiac disease such as CM	No	No CMs are not specifically part of the NPChUK. However, the Ministry is developing specific actions on CMs based on research identifying national and regional health needs including heart failure and CMs among a wide variety of conditions	No However, ESCAV mentions cardiomyopathies under a section dedicated to non-modifiable biological (i.e. genetic) risk factors	No	No	Partial NHS England Long-Term Plan has actions on some inherited CVDs; NHS England ‘Accelerating Genomic Medicine in the UK’ national genomics strategy includes inherited CVDs. Genetic testing for CM referred to in 2022 UK Government Office for Science project report, Genomics Beyond Health (2022). Scottish and Welsh plans above both specifically refer to inherited cardiac diseases, including CM
Expert centres exist for inherited cardiac diseases/CM (services include genetic testing)	Yes	Yes	Yes	Yes	Yes	Yes
Inherited cardiac disease/CM expert centres are organized/accredited/linked within a formal nationally/centrally co-ordinated network	No	No Will be tackled in the CM actions under development	Yes NHS Reference Centres, Services and Units (CSUR) cover the whole country and treat all patients equally with a multidisciplinary approach. The Ministry of Health grants CSUR accreditation through the Quality Agency via a verification process that includes genetic testing. Currently nine hospitals are accredited CSURs in familial heart diseases (including cardiomyopathies). See here and here	No	No	Yes NHS Genomic Medicine Service networks of excellence; inherited cardiac disease centres based on common national standards (see here) Network for Inherited Cardiac Conditions Scotland (NICCS) promotes multidisciplinary care to improve diagnosis and care according to evidence-based guidelines and audits practice and outcomes of centres against agreed national standards, working with patient representatives (also see here)
National governmental initiatives to promote implementation of/adherence to guidelines for screening and care for CM/inherited cardiac disease among cardiologists	No Dutch Medical Specialists’ Federation has established guidelines for inherited CVD/CM screening, but these are seemingly not included in Dutch Government’s register of official (treatment) guidelines (see here)	No Will be tackled in the CM actions under development	No	No	No	Yes For example, see networks above
National governmental policy for guideline-led/evidence-based reimbursement of inherited cardiac disease/CM genetic tests (for patients and family members)	Yes A wide spectrum of specialist medical care, and blood testing, theoretically qualifies for reimbursement within the ‘basic’ healthcare package (*Basispakket zorg*), but reimbursement decisions are at the discretion of individual (private) health insurers. Under basic health insurance schemes, patients contribute the first €385 (*Eigen risico*) of specialist medical costs	No Will be tackled in the CM actions under development	Yes Genetic testing and counselling are included in the Common Portfolio of Services of the NHS and thus are financed. The NHS finances various types of genetic tests (including diagnostic, carrier, prenatal, and pre-implantation) that meet the following requirements: having analytical and clinical validity based on scientific evidence, being of clinical utility, and having been previously assessed in relation to the ethical, social, legal, organizational, and economic implications of their inclusion in the public healthcare offer (see here)	Yes The costs of genetic testing and counselling are covered in full to patients referred within the NHS	Yes When prescribed by a doctor (see full list of reimbursed genetic testing here)	Yes This includes patients with CM/inherited CVD who have heart failure or implantable cardioverter defibrillator. All patients who undergo genetic testing for inherited CVD in England are entered in an NHS database
National governmental initiatives to promote inherited cardiac disease/CM family screening (among cardiologists, primary care physicians, nurses, public, etc.)	No	No Will be tackled in the CM actions under development	No	No	Not known	Yes, though limited (e.g. Genomics England Project to increase genetic testing for families who have lost someone due to SCD (see here)
National governmental initiative to promote health literacy and/or shared decision-making in cardiology clinical practice	No	No	No	No	Not known	Not known
National governmental initiatives to support networked data sharing infrastructures that include inherited cardiac diseases/CM	No	Yes P1, an electronic platform for digital medical records, enables public authorities, businesses, and private individuals to collect, analyse, and share digital resources related to medical issues, including CM. This platform is managed by the Centre of Healthcare Information Systems (CSIOZ), under the Ministry of Health	No	No	No	Yes (partial) National Institute for Cardiovascular Outcomes Research (NICOR) collects data and produces analysis to enable hospitals and healthcare improvement bodies to monitor and improve the quality of CVD care and patient outcomes
Direct national governmental funding dedicated specifically to CM/inherited CVD projects (within last 5 years)	Yes Governmental funding schemes via ZorgOnderzoek Nederland (ZonMw; Care Research Netherlands) and Nederlandse Organisatie voor Wetenschappelijk Onderzoek (NWO; Dutch Research Council	No	No	Yes Swedish Research Council funds research on CM, heart failure, and transthyretin amyloidosis	No	Yes National Institute for Health and Care Research (NIHR) has conducted multiple CM research projects in collaboration with other organizations

This table summarizes current governmental and health system-level policies, actions, and initiatives led, co-ordinated, or funded by the national health ministry, National Health Service, or national research funding body, as appropriate. We recognize that relevant additional initiatives led or funded by other stakeholders (including scientific and professional societies, patient organizations, and charities) exist in some countries.

ARVC, arrhythmogenic right ventricular cardiomyopathy; CM, cardiomyopathy; CVD, cardiovascular disease; HCM, hypertrophic cardiomyopathy; NCD, non-communicable diseases; NHS, National Health Service; SCD, sudden cardiac death.

**Figure 1:**
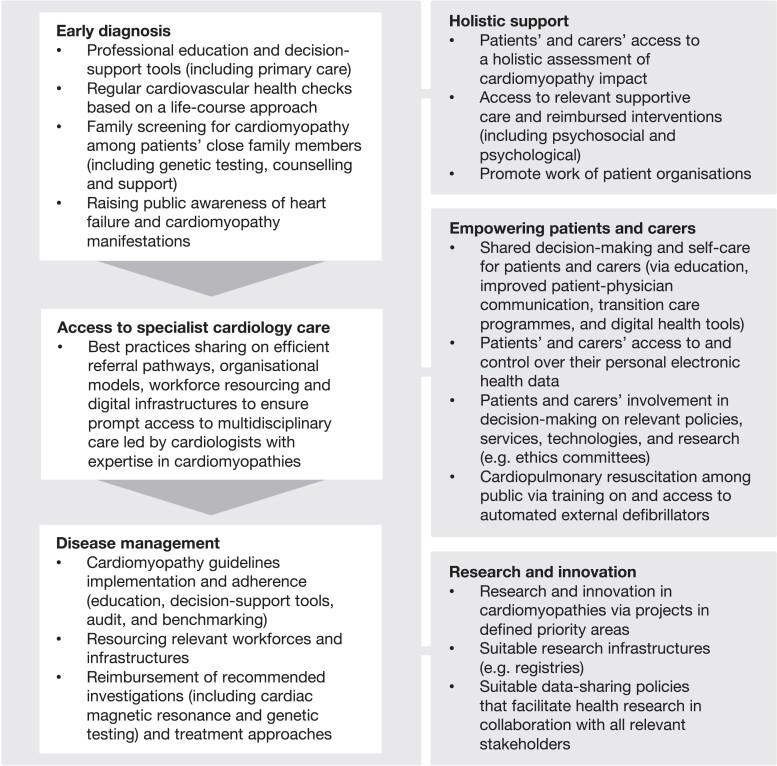
Summary of measures that national-level policy and health system decision-makers should implement to improve care and support for patients affected by cardiomyopathies and other inherited cardiovascular diseases. These include measures at key stages of the care pathway (diagnosis, referral, and disease management) and those that apply throughout all stages (holistic support for, and empowerment of patients and carers, and research and innovation)

Here, we offer national-level policy recommendations to help ensure that all patients and families affected by cardiomyopathies have access to guideline-led care (*Figure 1*). These are based on a *Policy Roadmap* developed by Cardiomyopathies Matter, a multi-stakeholder initiative launched in 2022 that aims to raise awareness of cardiomyopathies among policymakers and to ensure these health conditions are given the policy attention they warrant both at national and European levels (www.cardiomyopathiesmatter.eu).^2^

## Promoting efficient referral to expert care

All patients with cardiomyopathy should have prompt access to well–co-ordinated, multidisciplinary expert care—with ‘the family at its heart’.^2^ Fundamental to patients’ access are measures to ensure that primary care professionals (to whom patients with suspected cardiomyopathies may first present) have appropriate education and decision support tools and are supported to deliver regular cardiovascular health checks based on a life-course approach. Policy actions must then focus on the promotion of efficient referral pathways and organizational models that deliver expert care and the necessary workforces and digital infrastructures. Examples of national expert centre networks include those in France and Spain.^4^

## Realizing the value of genetics in optimizing patient care and early diagnosis

Genetic testing plays an increasing role in cardiomyopathy care and is recommended to optimize patient care and family screening (in conjunction with clinical screening).^2^ In addition, genetics could offer a path towards precision medicine for cardiomyopathies, with gene editing and gene therapy techniques already under investigation.^5^

A causative pathogenic variant can be identified in around 20%–60% of probands with the main four types of cardiomyopathy, i.e. hypertrophic cardiomyopathy, dilated cardiomyopathy, arrhythmogenic cardiomyopathy, and restrictive cardiomyopathy.^2^ Patients and families should have reimbursed access to genetic counselling by a suitably trained healthcare professional (before and after testing) to help them deal with the potential implications of these tests. Indeed, genetic counselling is recommended for everyone with an inherited or suspected inherited cardiomyopathy, regardless of whether they decide to undergo testing.

Promoting family screening is vital to drive early diagnosis. First-degree relatives of the patients should be offered clinical screening, with genetic testing offered if a disease-causing variant has been identified in the index patient.^2^ Genetic testing of family members can be cost-effective, by excluding the presence of genetic variants and thereby avoiding the need for follow-up.

Despite the increasing recognition of their impact, genetic counselling and testing are often underused,^2,6^ with substantial variations between and within countries, creating disparities in access. According to a systematic review and meta-analysis, only half to three-quarters of patients with hypertrophic cardiomyopathy and their relatives undergo genetic testing or cascade screening, and the uptake of genetic counselling is highly variable, despite being associated with high satisfaction and improved patient-reported outcomes.^7^ Other estimates suggest that overall genetic testing is performed in only 1%–2% of cardiomyopathy cases.^8^ Further variations exist in the availability of prenatal or pre-implantation genetic diagnosis to inform family planning.^9^

Policy actions necessary to ensure appropriate access to genetic testing and counselling (*Figure 1*) include measures to promote adherence to ESC clinical guidelines, policies and resourcing that support reimbursement based on guidelines, and sufficient resourcing to provide adequate levels of genetic workforce and laboratory capacity—something few countries achieve at present.^10^

Leveraging the value of genetics and other disease management advances also requires investment in systems for collection, analysis, and sharing of good-quality, harmonized, secure data to help optimize individual patient care, drive quality improvement, and support research and innovation.

## Supporting and empowering patients and families

Cardiomyopathies can present many challenges for patients and families, including morbidity, fears about sudden cardiac death, implications for daily activities, and concerns about inheritance. Holistic psychosocial support is therefore an important part of multidisciplinary cardiomyopathy care.^2^

Patients and carers play key roles in managing their conditions. National actions are necessary to empower them through measures that promote shared decision-making and self-care, including providing them with access and control over their own electronic health data (*Figure 1*). More broadly, empowerment efforts should extend to patients and carers’ involvement in decision-making regarding relevant healthcare policies, services, technologies, and research priorities. Policymakers should recognize and promote the role played by patient organizations in these matters.

National measures should also include campaigns to raise public awareness of inherited cardiovascular diseases, including cardiomyopathies, and associated symptoms and risks. Promoting public training in cardiopulmonary resuscitation and the use of automated external defibrillators is also vital, coupled with adequate and equitable access to these devices in public spaces.

## Conclusion

After decades of focus on coronary disease and its related risk factors, it is time to concentrate efforts and resources on genetic diseases, which are more prevalent than previously thought, disproportionately affect the young, and now are potential targets for an increasing number of innovative therapies. The Cardiomyopathies Matter initiative aims to take an important step in this direction by informing policy changes that improve outcomes for patients and families affected by cardiomyopathies, including inherited forms.

European Union–level measures already play a major role, including support for the GUARD Heart European Reference Network and research projects. We have recommended further EU-level measures, including a Cardiovascular Health Mission and Action Plan, leveraging the European Health Data Space, establishment of a European Cardiovascular Health Data Knowledge Centre, alignment of data sharing policies, and further research funding.^4^

We encourage cardiologists and allied healthcare professionals across Europe to collaborate with patient organizations and policymakers at all levels to promote national health policies that ensure all patients and families have access to guideline-informed care—Cardiomyopathies Matter initiatives are now in progress in many countries to this end.

## Notes

Cardiomyopathies Matter initiative contributors: Teofila (Tootie) Bueser (South East Genomic Medicine Service Alliance, Guy’s & St Thomas’ NHS Foundation Trust and North Thames Genomic Medicine Service Alliance, Great Ormond Street Hospital NHS Foundation Trust, London, UK), Ester Costafreda [Asociación para Pacientes y Familiares con Síndromes Arrítmicos relacionados con la Muerte Súbita (SAMS), Spain], Tomás Fajardo (Cardioalanza, Spain), Pablo Garcia-Pavia (Inherited Cardiac Diseases and Heart Failure Unit, Department of Cardiology, Hospital Universitario Puerta de Hierro and Centro Nacional de Investigaciones Cardiovasculares (CNIC), Madrid, Spain), Stefan Janssens (Department of Cardiovascular Diseases, University Hospitals Leuven, Leuven, Belgium), Hugo Katus (Department of Internal Medicine III—Cardiology, Angiology, and Pulmonology, Heidelberg University Hospital, Heidelberg, Germany), Stellan Mörner (Department of Public Health and Clinical Medicine, Umeå University, Umeå, Sweden), Iacopo Olivotto (Department of Experimental and Clinical Medicine, University of Florence and Meyer University Children Hospital, Florence, Italy), Matteo Pinciroli [Cardiomyopathy Patient Council, Global Heart Hub and Italian Association for the Study and Research of Cardiomyopathies (AICARM)], Sean Taylor (World Heart Federation, Geneva, Switzerland), Maria T. Tome Esteban (Cardiovascular Clinical Academic Group, Inherited Cardiac Condition Service, St George’s Hospital NHS Foundation Trust and St George’s University of London, London, UK), Patricia Tiramani (Global Heart Hub, Galway, Ireland), Jean-Noël Trochu (Thorax Institute, Heart Failure and Cardiomyopathies Unit, University Hospital, Nantes, France), Rogier Veltrop (LMNA Lab Mechanobiochemistry and Cardiac Cell Signalling, Cardiovascular Research Institute Maastricht, Maastricht University, and LMNA Cardiac Foundation, Maastricht, The Netherlands), Lee Baker (Cardiomyopathies Matter Secretariat, Brussels, Belgium).
