# Correction to: Family matters: health policies to tackle cardiomyopathies across Europe

**DOI:** 10.1093/eurheartj/ehae898

**Published:** 2024-12-19

This is a correction to: Iacopo Olivotto, Cardiomyopathies Matter Initiative, on behalf of the, Family matters: health policies to tackle cardiomyopathies across Europe, *European Heart Journal*, Volume 46, Issue 1, 1 January 2025, Pages 6–14, https://doi.org/10.1093/eurheartj/ehae419

In the originally published version of this paper, there were a number of errors present in Table 1:

- The row labelled “National governmental funding for inherited cardiac disease/CM research projects (within last 5 years)” should have been labelled as “Direct national governmental funding dedicated specifically to CM/inherited CVD projects (within last 5 years)”.
- In the row labelled “National governmental policy for guideline-led/evidence-based reimbursement of inherited cardiac disease/CM genetic tests (for patients and family members)” the UK column was given as “Yes Genetic testing available free of charge to patients on NHS upon referral from hospital specialist”. This should have been given as “Yes This includes patients with CM/inherited CVD who have heart failure or implantable cardioverter defibrillator.

All patients who undergo genetic testing for inherited CVD in England are entered in an NHS database”

- In the row labelled “National governmental initiatives to support networked data sharing infrastructures that include inherited cardiac diseases/CM” the UK column was given as “Yes”. This should have been given as “Yes (partial)”.

In the final row labelled “National governmental funding for inherited cardiac disease/CM research projects (within last 5 years)” the following errors were present:

- The row label was incorrect and should have been given as “Direct national governmental funding dedicated specifically to CM/inherited CVD projects (within last 5 years)”.
- The Netherlands column was given as “No”. This should have been “Yes Governmental funding schemes via ZorgOnderzoek Nederland (ZonMw; Care Research Netherlands) and Nederlandse Organisatie voor Wetenschappelijk Onderzoek (NWO; Dutch Research Council”
- The Spain column was given as “Yes (via Spanish National Center for Cardiovascular Research (CNIC) Inherited Cardiomyopathies research group)”. This should have been given as “No”.
- The UK column was given as “Yes National Institute for Health and Care Research (NIHR) conducts research in collaboration with other organizations”. This should have been “Yes National Institute for Health and Care Research (NIHR) has conducted multiple CM research projects in collaboration with other organizations”

These errors have been corrected in the paper.

